# An Algorithm for the Diagnosis and Management of Severe Asthma in Children and Adolescents

**DOI:** 10.1111/all.70027

**Published:** 2025-08-29

**Authors:** Eckard Hamelmann, Bianca Schaub, Susanne Lau, Leonard B. Bacharier

**Affiliations:** ^1^ Department of Paediatrics, Children's Center Bethel, Bielefeld University Hospital OWL, University of Bielefeld Germany; ^2^ Department of Pulmonary and Allergy, Dept. of Paediatrics, Dr von Hauner Children's Hospital University Children's Hospital, Ludwig‐Maximilians‐University Munich Germany; ^3^ Comprehensive Pneumology Center (CPC‐M), LMU Munich, Member of the German Center for Lung Research (DZL), and German Center for Child and Adolescent Health (DZKJ) Dr von Hauner Children's Hospital, LMU Munich Germany; ^4^ Department of Pediatric Respiratory Medicine, Immunology and Critical Care Medicine Charité Universitätsmedizin Berlin Berlin Germany; ^5^ Department of Pediatrics Monroe Carell Jr. Children's Hospital at Vanderbilt University Medical Center Tennessee USA

**Keywords:** asthma, asthma treatment, biologics, paediatrics, severe asthma

1

DTA is present when good control cannot be achieved for ≥ 6 months under step 4 therapy (medium‐dose inhaled corticosteroids [ICS] + long‐acting β2 agonist or equivalent) owing to modifiable factors such as poor adherence, faulty inhalation technique, trigger exposure or untreated comorbidities.

**FIGURE 1:**
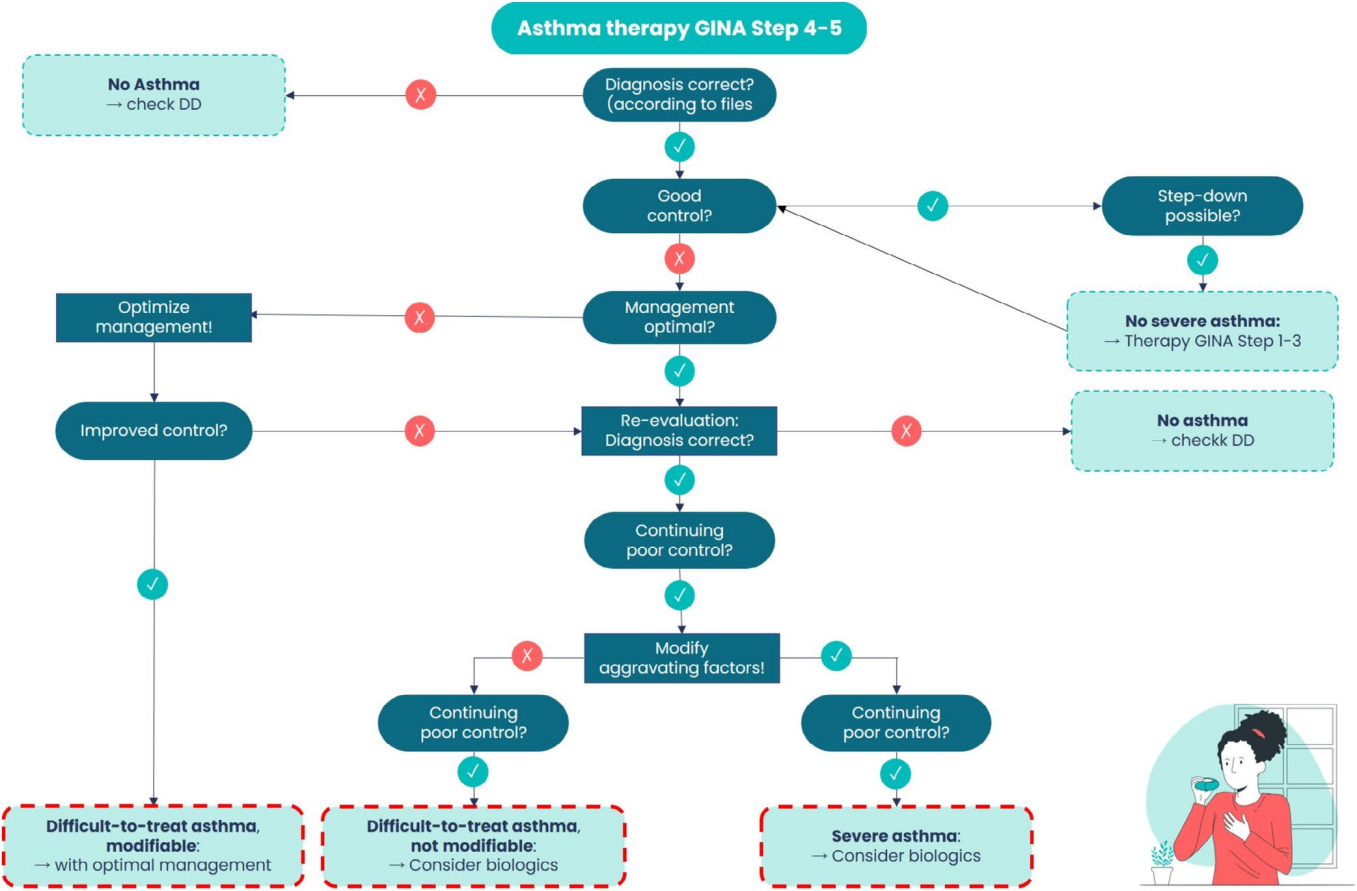
Diagnostic algorithm distinguishing difficulttotreat from severe asthma in children and adolescents (adapted from 3). The flow chart starts with every patient who is receiving high‐dose controller therapy (Step 4: Medium‐dose ICS + LABA; step 5: Highdose ICS + LABA/LAMA/biologic). Each decision node asks a single yes/no question and directs the clinician towards one of five mutually exclusive outcomes. Is the asthma diagnosis correct? Objective lung function variability, bronchodilator reversibility and, where needed, provocation testing or imaging, are reviewed. A negative answer exits the pathway at ‘No asthma → Evaluate differential diagnoses’ such as cystic fibrosis, primary ciliary dyskinesia or postinfectious bronchiolitis obliterans. Is asthma well controlled? Using (c) ACT/ACQ, exacerbation history and spirometry, good control triggers a stepdown trial. Sustained stability for ≥ 3 months leads to ‘No severe asthma → Continue GINA steps 1–3’. Loss of control during stepdown returns the patient to the main branch. Is current management optimal? Electronic adherence data, direct inhaler technique observation, medications use e.g., SABA overuse or NSAIDS, trigger avoidance and treatment of comorbidities (allergic rhinitis, atopic dermatitis, GERD, obesity, etc.) are assessed. If deficits are identified, the branch ‘Optimise management!’ is activated. Restoration of stable control following these interventions defines—‘Difficulttotreat asthma, modifiable’. After optimisation, does poor control persist? If yes, the diagnosis of asthma is reconfirmed, and the algorithm asks: Can aggravating factors be modified? Persistent allergen exposure, environmental tobacco smoke or psychosocial stress that cannot realistically be eliminated categorises the patient as—‘Difficulttotreat asthma, not modifiable – consider biologics’. Aggravating factors modified? Continued uncontrolled disease after modification of aggravating factors on high‐dose therapy fulfils NVL 5.0, S1 2025 and GINA 2025 criteria for ‘Severe asthma – consider biologics’. For the three lower boxes, national guidance recommends referral to a paediatric severe asthma centre for comprehensive biomarker profiling (blood eosinophils, FeNO, total/specific IgE) and selection of the most appropriate biologic, as detailed by Bacharier 2025. This structured pathway ensures that patients receive the least burdensome, yet evidence‐based therapy while avoiding unnecessary systemic corticosteroid exposure.

**FIGURE 2:**
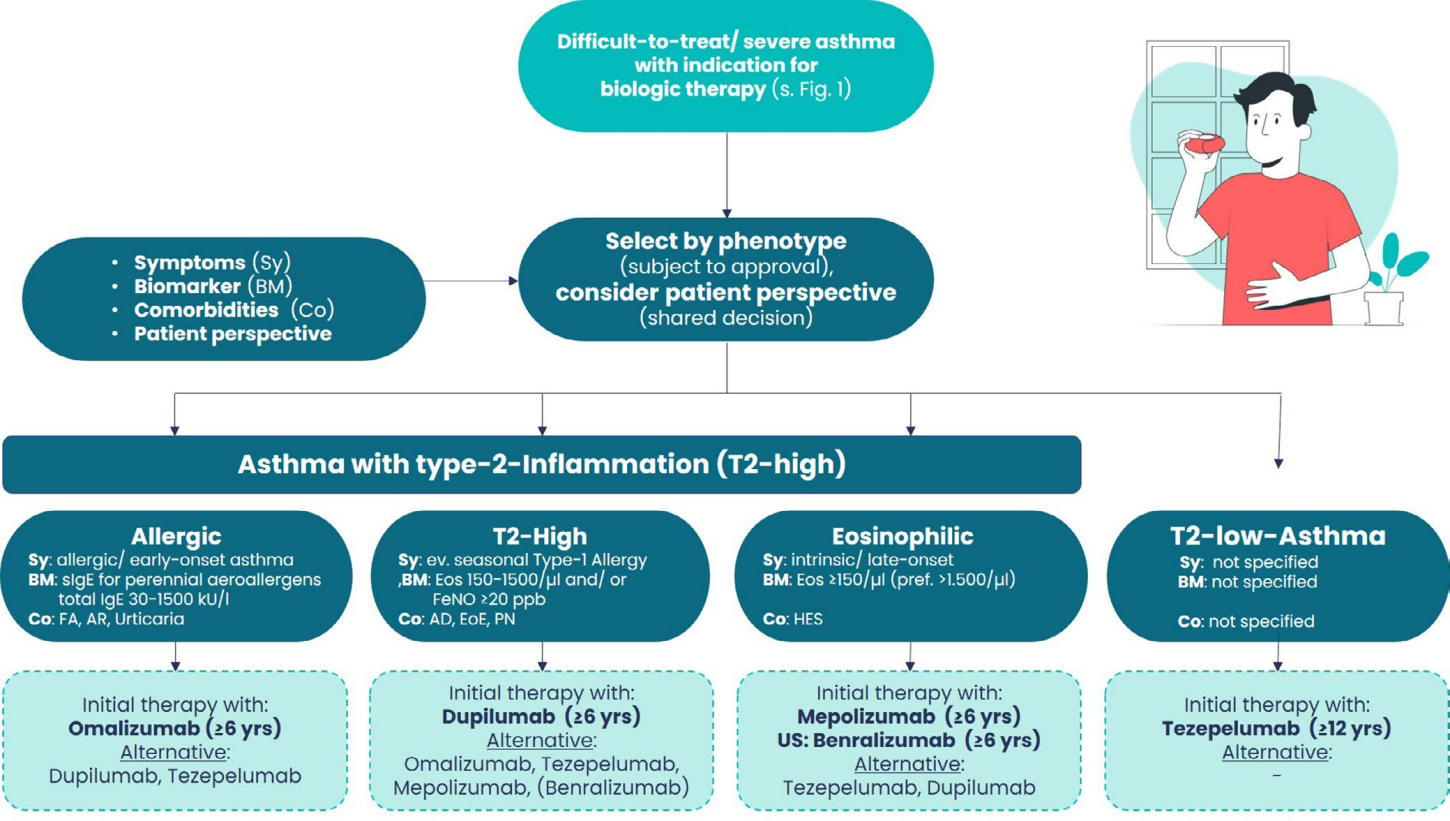
Phenotype‐based algorithm for the initial selection of biologic therapy in children and adolescents with an indication for add‐on treatment (adapted from 3). Figure 2 translates the two downstream branches of Figure 1—*‐*‘Difficulttotreat asthma, not modifiable – consider biologics’ and ‘Severe asthma – consider biologics’—into a structured decision tool that aligns current guideline recommendations with the paediatric evidence base and the patient's perspective (shared decision). The entry point is any patient whose disease remains uncontrolled on high‐dose ICS/LABA (± LAMA) after optimisation/addressing of adherence, comorbidities and aggravating factors. A four‐component assessment is performed: Symptoms (Sy): Clinical history distinguishes *allergic/early‐onset* versus *intrinsic/late‐onset* disease and screens for exacerbation patterns. Biomarkers (BM): Serum total IgE, perennial allergen‐specific IgE, blood eosinophils (Eos) and fractional exhaled nitric oxide (FeNO) are measured as surrogates of type 2 inflammation; cut‐offs reflect those used in pivotal trials (e.g., IgE 30–1500 kU L^−1^, Eos ≥ 150–1500 μL^−1^, FeNO ≥ 20 ppb). Comorbidities (Co): Treatable traits such as food allergy (FA), allergic rhinitis (AR), atopic dermatitis (AD), eosinophilic oesophagitis (EOE), nasal polyps (PN) or chronic urticaria help refine phenotype and may favour one biologic over another. Patient's perspective: Include the child's and family's perspective in the decision. Consider the specific application, costs, and availability of the various biologics. The algorithm then allocates the patient to one of four inflammatory patterns: Type2high allergic asthma (early‐onset/perennial sensitisation)‐—omalizumab is recommended as firstline; dupilumab or tezepelumab are listed as alternatives/secondline for partial or nonresponders, consistent with the 2025 German S1 guideline and Bacharier 2025. Type2high asthma with moderate eosinophilia or raised FeNO—dupilumab is preferred owing to its dual IL4/IL13 blockade and efficacy across IgE strata; omalizumab, mepolizumab (benralizumab), and tezepelumab are secondary options. Type2high eosinophilic (intrinsic/lateonset) asthma—mepolizumab is advised first; benralizumab is an equivalent choice where licensed. Nonresponders may switch to tezepelumab or dupilumab. Type2low asthma—characterised by absent type2 biomarkers; tezepelumab, which targets TSLP upstream of the inflammatory cascade, is recommended; no evidence‐based secondline exists yet. A 16‐ to 24‐week trial period with predefined response criteria (≥ 50% reduction in severe exacerbations, ≥ 10 ppb FeNO drop or ≥ 0.1 L FEV₁ gain) determines whether to continue, switch or escalate therapy. The stepwise, phenotype‐anchored approach maximises the likelihood of clinical benefit, minimises systemic steroid exposure and aligns with precision medicine principles endorsed by NVL 5.0 and international consensus documents.

SA is diagnosed only after these factors are corrected *and* the diagnosis of asthma is reconfirmed, yet uncontrolled asthma or the need for high‐dose ICS or medium‐dose ICS plus another controller (long‐acting beta‐agonists and/or long‐acting muscarinic antagonist) or biologic therapy (Step 5/6) persists.

This definition is consistent with the 2025 German S1 guideline, the 2024 National Asthma Guideline and GINA 2025 [1, 2, 3].

1. Confirm the diagnosis—exclude alternative or coexisting diseases (e.g., cystic fibrosis, primary ciliary dyskinesia, immunodeficiency, postinfectious bronchiolitis obliterans and inducible laryngeal obstruction) by history, lung function, imaging and, when indicated, bronchoscopy.
2. Assess control and risk—use validated tools (cACT/ACT, ACQ) and document recent exacerbations, systemic steroid bursts and lung function impairment.
3. Optimise baseline care—verify therapy adherence and inspiratory flow, coach inhaler technique, prescribe a written action plan, offer structured asthma education and consider pulmonary rehabilitation and MART with low‐dose ICS formoterol as reliever where licensed.
4. Address modifiable aggravating factors—reduce allergen and tobacco exposure, manage psychosocial stress, correct inhaler technique, ensure vaccination and treat chronic infections.
5. Identify and treat comorbidities—especially allergic rhinitis, atopic dermatitis, food allergy, chronic rhinosinusitis, gastrooesophageal reflux, obesity and sleep apnoea.
6. Reevaluate—if control remains poor despite ≥ 3 months of optimised Step 5 therapy, proceed to phenotype assessment and targeted biologics, preferably in a paediatric severe asthma centre.

Most paediatric SA is type 2 high. Persistent elevation of biomarkers predicts both higher exacerbation risk and superior biologic response. Integration of clinical phenotype with biomarker pattern, age, dosing logistics (such as weight‐based dosing of omalizumab and application forms and intervals of biologics) and relevant comorbidities allows precision therapy [3, 4]:

- Biomarkers—peripheral blood eosinophils (≥150 cells/μl), FeNO (≥20 ppb) and/or total/specific IgE and aeroallergen sensitisation support a type 2‐high phenotype.
- Phenotype allocation—(1) type‐2‐high, with sub‐phenotypes of (1.1) allergic, (1.2) t2‐high, (1.3) eosinophilic intrinsic/nonallergic, [2] type2‐low.
- Selection of biological—*Omalizumab* for IgE‐mediated allergic asthma with perennial sensitisation (age ≥ 6 y) and/or food allergy or chronic spontaneous urticaria;
  • *Mepolizumab* (age ≥ 6 y) or *Benralizumab* (age ≥ 6 y only in USA, ≥12 y elsewhere) for eosinophilic asthma (first line if eos >1500/μl);
  • *Dupilumab* for type 2‐high asthma with elevated FeNO or eosinophils and/or coexisting atopic dermatitis (age ≥ 6 y);
  • *Tezepelumab* for uncontrolled asthma irrespective of IgE/eosinophils (age ≥ 12 y).

A response assessment at 16–24 weeks should include symptoms, exacerbations, lung function and biomarker trends. Targets are ≥ 50% exacerbation reduction, ≥ 0.1 L FEV_1_ gain or relevant improvement in cACT/quality of life [5]. Partial responders may continue; non‐responders require adherence audit, phenotype reassessment or switch to an alternative pathway.

Quarterly review is recommended: symptom score, exacerbation record, spirometry with bronchodilator, growth surveillance and health‐related quality of life (e.g., PAQLQ). FeNO every 6 months helps to gauge type 2 activity and biologic response. Check also for modifiable factors and limitations of adherence in order to achieve the best control of symptoms and full participation in daily activities. Sustained control allows gradual ICS step‐down; relapse prompts reinstatement at the last effective dose.

NVL 5.0 advises referral to a paediatric pulmonology centre before Step 5 escalation and prior to biologic initiation, and recommends rehabilitation to optimise adherence before further intensification [1]. National registries such as German AsthmaNet support benchmarking and equitable biologic access while promoting ‘steroid stewardship’ to minimise cumulative systemic‐steroid burden [2, 3].

## Conflicts of Interest

**Eckard Hamelmann:** ALK, AstraZeneca, DBV Technologies, GSK, Regeneron Pharmaceuticals Inc., Sanofi—speaker/consultant fees; BMBF, BMG, MAGS, G‐BA—research support; Wolff—data and safety monitoring board. **Bianca Schaub:** GSK, Novartis, AstraZeneca, Sanofi—speaker/consultant fees; German Center for Child and Adolescent Health (DZKJ; LMU/LMU Klinikum: 01GL2406A), DFG—research support; Sanofi—Data Safety Monitoring Board. **Susanne Lau:** Received honoraria for lectures and advisory boards from Sanofi‐Aventis, AstraZeneca, Allergopharma, ALK, GSK, Leo Pharma, Leti, Viatris, Lilly, and DBV. **Leonard B. Bacharier:** AstraZeneca, GSK, OM Pharma, Regeneron Pharmaceuticals Inc., Sanofi—speaker/consultant fees; NIH, OM Pharma, Sanofi, Vectura—research support; Aravax, AstraZeneca, Cystic Fibrosis Foundation, DBV Technologies—data and safety monitoring board.

## Data Availability

All data supporting the findings of this study are available within the paper.
